# Fungal rhinosinusitis in patients with chronic sinusal disease

**DOI:** 10.1016/S1808-8694(15)31237-4

**Published:** 2015-10-20

**Authors:** Celso Dall'Igna, Bruno Carlos Palombini, Fabio Anselmi, Elisabeth Araújo, Daniela Pernigotti Dall'Igna

**Affiliations:** Ph.D. in Medicine, UFRGS, Joint Professor of Otorhinolaryngology, UFRGS, Head of the Service of Otorhinolaryngology, Hospital de Clínicas de Porto Alegre; Faculty Professor of Pneumology, UFRGS, Head of the Service of Pneumology - Pavilhão Pereira Filho, Santa Casa de Misericórdia de Porto Alegre; Otorhinolaryngologist; Master and Ph.D. in Medicine, Professor of Post-graduation in Pneumology, UFRGS. Otorhinolaryngologist; Resident Physician in Otorhinolaryngology, UFPR. Federal University of Rio Grande do Sul

**Keywords:** sinusitis, mycosis, fungus, nose

## Abstract

**Aim:**

Fungal rhinosinusitis in patients with chronic sinusal disease study. In the past decades, there has been an increase in fungal infections, and fungal rhinosinusitis (FRS) has been diagnosed more frequently. Knowing the fungal flora, its prevalence and symptomatic presentation in patients with chronic rhinosinusitis (CRS) will allow a better understanding of this disease, permitting a correct diagnosis, and treatment and formulating its prognosis.

**Study design:**

clinical retrospective with transversal cohort.

**Material and Method:**

62 patients diagnosed with FRS were selected among 890 cases of CRS undergoing endoscopic surgery. We assessed clinical history otolaryngologic examination with nasal videoendoscopy, CT scan, microbiologic and histopatologic tests.

**Results:**

The prevalence of FRS was 6.7% and the fungal type most frequently found was the gender Aspergillus. Fungal ball was found in more than half the cases, and allergic fungal rhinosinusitis (AFRS) in more than one third of the patients. Evolution after endoscopic sinus surgery was more favorable in patients with fungus ball, with a minor number of recurrences.

**Conclusions:**

The symptomatic evolution following endoscopic surgery was more favorable in patients with fungal ball, who require a lower number of re-interventions.

## INTRODUCTION

Fungi are present in most ecosystems and there are over 20,000 species already identified. In the past two decades medicine has witnessed the increase in fungal infections in humans, caused by over 250 different species, as a result of the increase in use of immunosuppressant drugs for the treatment of cancer and control of transplanted organ rejection, infections by human immunodeficiency virus (HIV), diabetes mellitus, use of vascular and urinary catheters and use of broad spectrum antibiotics. From simple candidiasis to mortal aspergillosis, mycoses are frequently present in all levels of daily medical practice.

Fungal rhinosinusitis (FRS) has been referred in the medical literature for over two centuries. However, for the past 25 years it has received the due attention, with increase in diagnostic suspicion and improvement in laboratory techniques for detection of fungi. Its classification, based on immune relation between fungi and their host and level of mucosa invasion, is important in the selection of an effective treatment and for the definition of the prognosis. The knowledge of this type of fungal flora, its prevalence, symptomatic presentation, aspects of the physical examination and supplementary tests in patients with chronic rhinosinusitis (CRS) will enable better understanding of the disease, raising awareness of the involved physicians for appropriate diagnosis and treatment.

The present study was performed to define the prevalence of fungi infections in patients with CRS submitted to surgical treatment, its classification according to FRS and clinical presentation followed by progression after the application of treatment.

## PATIENTS AND METHODS

### Study Design

The present study comprised a cohort of patients with CRS submitted to surgical approach of paranasal sinuses using endoscopic method, between January 1988 and December 2002 in Porto Alegre, RS, with the diagnosis of FRS. The studied factors were the classification of patients and the outcome was their progression.

### Criteria for inclusion and exclusion

We assessed 890 patients with CRS submitted to paranasal sinuses endoscopic surgical procedure and we selected 62 patients with diagnosis of FRS, confirmed by direct mycological and/or culture and/or clinical pathology analysis.

We considered the patients with CRS and signs and symptoms of inflammation of paranasal sinuses that persisted for more than 12 weeks, associated with the documented affections by imaging techniques after at least 4 weeks of appropriate clinical treatment^1^.

We excluded patients that did not agree to participate in the study or sign the informed consent term, those that had not been postoperatively followed up for at least 6 months and those that did not meet the above-mentioned criteria.

### Method of sample collection

Middle meatus or paranasal sinuses secretions were collected unilaterally, under endoscopic view, with aspirator sterilized in autoclave at 2mm diameter coupled in a collection recipient Specimen Trap model 076-0490 (Sherwood Medical, St. Louis, USA).

### Patients' assessment

Anamnesis - It comprised general clinical history and specific history directed to ENT affections by assessing the following data: nasal obstruction, rhinorrhea, nasal pruritus, sneezing, post-nasal dripping, epistaxis, olfaction affections, oropharynx complaints, headache, hoarseness, cough, asthma, and expectoration.

Otorhinolaryngological examination - The exam included nasal videoendoscopy, performed by rigid endoscope of 4mm and 30° and 0° angle or 6mm flexible endoscope, under local anesthesia with cotton ball soaked in neotutocaine at 2% and vasoconstriction with oxymetazolin when necessary. We observed the following data: nasal septal deviation, aspect of medium and inferior turbines, mucosa color, presence and type of secretion, polyposis, polypoid degeneration and middle meatus status.

Radiological study - We performed paranasal sinuses CT scan, without intravenous contrast at axial and coronal plans, with patients in dorsal decubitus. Presence of microcalcifications and metallic density image inside the paranasal sinuses were considered as suggestive of presence of fungi^2^^,^^3^.

Microbiological exam - Clinical sample were collected during the surgical procedure and placed in Stuart transportation medium (Starplex Scientific, Ontario, Canada) for the cultivation of aerobe microorganisms, in a broth of thioglycolate for the cultivation of anaerobes. The material was referred for analysis within 1 hour from collection.

For aerobe culture, the material was prepared in plaques containing agar medium McConkey (Difco, Detroit, USA or Becton Dickinson, Maryland, USA) and Triptycase Soy agar (Difco, Detroit, USA) enriched with 10% goat blood (agar blood) and incubated at 37o C for 24 hours. If there was no bacterial growth, the medium was reincubated for 24 hours more hours before it was released as negative.

The cultivation for anaerobe germs was performed through preparation in goat blood agar and the base was agar blood brucella (Difco, Detroit, USA) and bacteroid bile esculin agar, with incubation for up to 72 hours in atmosphere of anaerobiosis provided by systems Gaspak (Becton Dickinson, Maryland, USA), Anaerocult (Merck SA, Brazil) or Anaerobac (Probac, Sao Paulo, Brazil). Thioglycolate broth was used as back up for cultivating anaerobes in case of suspicion of presence of germs in the sample (estimated by Gram method) and absence of plaque growth. After isolation and confirmation of anaerobe germ, the identification of the microorganism was made by using API system for anaerobe germs (Bio Merieux, France).

Mycological analysis was performed through direct material examination between the lamina and the laminula and the culture of the material in medium Sabouraud with or without cloramphenicol and cyclohexamide. Incubation was at 25o-35 C, and cultures were observed up to 20 days before release as negative for fungi. The identification of fungi and yeasts were made from microscopic morphology and use of commercial kit to identify the yeasts (Systems API, Bio-Merieux, France), respectively.

Histopathological exam - The samples of the mucosa of the involved sinus, according to the histopathological findings were classified as:

a) nonspecific chronic inflammation (edema with thickness of mucosa lamina propria, lymphoplasmocytarian infiltrate and perivascular fibrosis);
b) suppurative chronic inflammation (previous characteristics with neutrocytarian infiltrate);
c) allergic chronic inflammation (chronic inflammation with eosinophilic infiltrate);
d) chronic inflammation with fungal invasion (visualization of filamented and segmented hyphae with staining by PAS and Grocot methods).

### Classification of patients

Patients were classified according to type of FRS, according to surgical and histopathological findings, such as fungal ball, saprophyte infection, invasive FRS, AFRS and indolent FRS, as provided by Ferguson criteria^4^:

a. fungal ball: characterized by entangled hyphae in the paranasal sinuses, without tissue invasion and minimum mucosa inflammation reaction;
b. saprophyte infestation: asymptomatic presence of visible fungi in mucosa crusts of the nose or the paranasal sinuses, without mucosa invasion;
c. invasive fungal rhinosinusitis: fungal infection with tissue invasion at histopathological exam, with or without vascular invasion;
d. allergic fungal rhinosinusitis: presence of mucus with numerous eosinophils, Charcot-Leyden crystals, rare hyphae and mucosa inflammation reaction, without fungi invasion;
e. indolent or chronic invasive fungal rhinosinusitis: presence of fungi with absent or minimal vascular invasion.

### Patients' follow up

The follow-up of patients was performed by the authors using anamnesis, complete ENT examination and nasal endoscopic exam. The follow-up was performed for a minimum of 6 months.

### Statistical analysis

To compare the proportions, we used the chi-square test, with correction of Yates when the number was one, and Fischer exact test if the expected number for some specific characteristic was below 5 patients.

Symptoms were compared by using the score scale with t test for matched samples (parametric data) and T Wilcoxon test (non-parametric data for matched samples), and ANOVA (analysis of variance) for repetitive measurements for mean comparisons.

For α = 0.05 and β = 0.20 and to identify the differences of exposure and control of at least 20% to 25%, we estimated a minimum sample size of 60 patients per group (total = 120). The acceptable error was 5% (p < 0.05).

The project was approved by the Research Ethics Committee of Hospital de Clínicas de Porto Alegre.

## RESULTS

Out of a total of 890 nasosinusal surgical procedures, we selected the cases in which we detected the presence of fungi identified by laboratory exams, which are the object of this study.

We analyzed the data of 62 patients (6.7%) with mean age of 40.6 years (ranging from 8 to 81 years, median of 45.4 years), 25 male and 37 female patients, without statistically significant difference (p = 0.13, t Student test). Only one patient was Black, and all the others were Caucasian.

Signs and symptoms found are summed up in Table 1. The duration of symptoms ranged from 3 months to over 5 years.

**Table 1:** Preoperative clinical manifestation of studied patients.

Signs and symptoms	n	%
nasal obstruction	57	92
nasal secretion	55	89
post-nasal dripping	51	82
cough	43	69
allergic rhinitis	30	48
olfaction affections	21	34
asthma	21	34
sore throat	20	32
intolerance to aspirin	14	22
fatigue	14	22
facial pain	9	15

The most frequent comorbidities were rhinitis in 33 patients (53.2%), bronchial asthma in 21 patients (33.8%), hypertension in 16 (25.8%), and aspirin intolerance in 14 (22.5%). There were fewer cases of repetitive bronchopneumonia (7 patients), bronchiectasia (6 patients), hepatitis (4 cases), organ transplantation and primary immunodeficiency (3 patients), diabetes mellitus and leukemia (2 patients) and isolated cases of cystic fibrosis, tuberculosis and hypophysis tumor. As to previous use of medication, 59 patients (95.1%) had used broad-spectrum antibiotic in the month before the surgery, 37 (59.6%) had used topical nasal corticoids, 25 (40.3%) had used oral corticoids, and 6 (9.7%) had received some type of immunosuppressant drugs that were not corticoids.

Only 11 patients (18.6%) had been previously submitted to nasosinusal surgery: 3 to endoscopic polypectomy, 4 to sinusotomy, 1 to septoplasty, 1 to rhinoplasty, 1 to septoplasty and polypectomy and 1 to turbinectomy and sinusotomy.

Nasal endoscopic findings were: nasal secretion in 57 patients (91.9%), divided into 29 (46.7%) yellowish color, 23 (25%) greenish, 4 were brown and 1 was black; medium meatus obstruction in 47 (75.8), polyposis in 25 (40.3%), bilateral in 10 (16.1%) and unilateral in 15 (24.1%), lower conchae hypertrophy in 17 (27.4%) and adenoid hypertrophy in 3 (4.8%).

All patients presented some type of opacification of paranasal sinuses CT scan and all other findings are listed in Table 2.

**Table 2:** CT scan findings.

	n	%
Opacification	62	100,0
Middle meatus obstruction	49	79,0
Thickness or bone sclerosis	34	54,8
Microcalcifications	28	47,4
Metallic density images	18	29,0
Bone erosion	16	25,4
Sinus expansion	11	17,7

Mycological culture exam was obtained during surgery and showed gender Aspergillus as the most frequent, followed by Candida and Penicilium (Table 3).

**Table 3:** Types of fungi identified in the mycological culture.

	n	%
*Aspergillus sp.*	30	48,3
*Candida sp*	11	17,7
*Penicillium*	5	8,0
septated and ramified hyphae	5	8,0
*Alternaria sp.*	3	4,8
others	8	12,8

Bacteriological examination indicated associated bacterial infection in 47 patients with growth of S. aureus in 15 cases (31.9%), P. aeruginosa in 10 (21.2%) and Haemophillus sp. in 4 (8.5%). In 10 patients (16.9%) there was no bacterial growth and in 2 the exam was not performed.

Polypectomy was performed in 23 patients, in 6 unilateral and in 17 bilateral cases.

Definite histopathological exam showed nonspecific chronic inflammation in 25 cases (40.3%), allergic chronic inflammation in 21 cases (33.8%), suppurative chronic inflammation in 12 (19.3%), and fungal invasion in 4 (6.4%). As to FRS classification, we observed fungal ball in 33 patients (53.2%), AFRS in 24 (38.7%) and indolent FRS in 3 (4.8%). In 2 patients, fungi were considered saprophyte. There was no case of invasive fungal rhinosinusitis (Table 4).

**Table 4:** Types of fungal rhinosinusitis.

	n	%
Fungal ball	33	53,2
Allergic fungal rhinosinusitis	24	38,7
Indolent fungal rhinosinusitis	3	4,8
Saprophyte infection	2	3,2
Invasive fungal rhinosinusitis	0	–
Total	62	100.0

Among the cases classified as fungal ball, nasal obstruction was present in 30 (90.9%), posterior nasal secretion in 29 (87.8%), and topical corticoids in only 9 (27.3%). Cough was the complaint in 19 cases (57.5%), intolerance to aspirin was detected in only 1 case (3.5%) and 6 (18.2%) had repetitive pneumonia. Polyps were seen in only 2 cases (7.1%) and middle meatus obstruction in 7 (21.2%).

The patients with AFRS presented as a whole nasal obstruction, posterior nasal secretion and previous use of topical corticoids and systemic antibiotics. Cough was the complaint in 14 (58.3%) cases, 12 (50%) referred intolerance to aspirin, and only 5 (20.1%) had previous pneumonia. Surgical findings evidenced multiple polyps in 22 patients (19.6%), medium meatus obstruction in 21 (87.5%) and microcalcifications in 20 (81.8%) of the cases. The most frequent type of fungus was Aspergillus (8 cases).

Table 5 shows the comparison of preoperative findings between these 2 groups.

**Table 5:** Preoperative findings according to type of fungal rhinosinusitis.

Symptom	Type of RFS		Significance
	RSFA	Fungal ball	
Nasal obstruction	100	91	NS*
posterior secretion	100	88	NS*
Topical corticoid	100	29	p < 0,001**
Systemic Antibiotic	100	94	NS*
Allergic rhinitis	81	36	NS*
Asthma	77	11	p < 0,001**
Cough	58	57	NS*
Intolerance to AAS	50	3	p < 0,02**
Repetitive Bronchopneumonia	20	18	NS*

p = Pearson p value for Chi-square test.

* NS - not significant (p > 0.05)

** Yates correction of Chi-square

There was no statistically significant difference when we compared mean age (p = 0.13, t Student test) and gender of the group with diagnosis of AFRS and fungal ball. Preoperative symptoms were statistically analyzed after placed in a scale and they were globally compared between the two diagnoses. The scores of the group using t test for independent samples did not show any difference between them (p = 0.715; CI 95%: −1.11 to 1.61).

Conversely, the use of topical corticoids, history of previous intolerance to aspirin and bronchial asthma, when individually analyzed, were the only ones that showed statistically significant difference in the comparison of preoperative symptoms between the two groups, and it was more frequent in the patients with diagnosis of AFRS. The presence of polyps in the preoperative clinical exam in patients with RSFA (78.2%) was statistically greater (p < 0.001, t Student test) than in the ones of fungal ball diagnosis (6.0%).

Comparing the radiographical studies with CT scan in both groups, the finding of obstruction of the middle meatus did not show statistically significant difference (chi-square test). The other findings, such as presence of bone erosion, were found in 12 patients (50%) with AFRS and in 4 (12.1%) with fungal ball (p < 0.001), microcalcification in 19 (79.1%) and 10 (30.3%) (p < 0.001), and mucosa thickness in 8 (33.3%) and 2 (6.1%) patients (p = 0.02), respectively, evidencing a statistically significant difference between the groups.

As to type of fungus, we found there were no statistically significant differences in cultures.

Table 6 shows the comparison of surgical findings between the patients with diagnosis of fungal ball and those with AFRS.

**Table 6:** Transoperative findings in cases of allergic fungal rhinosinusitis and fungal ball.

Transoperative findings	RFS Type		Significance
	RSFA	Fungal ball	
Middle meatus obstruction	88	24	p < 0,001
Microcalcification	79	33	p < 0,02**
Polyps	91	6	p < 0,001*

p = Pearson p value for Chi-square test.

* Fisher exact test

** Yates correction of Chi-square

Postoperative follow-up was on average 19.4 months, with standard deviation of 6.3 months. Twenty patients (32.2%) were followed up for 6 months to 1 year, 14 (22.6%) from 1 to 2 years, 6 from 3 to 4 years (9.7%), 7 (11.2%) from 4 to 5 years, and 15 (24.2%) were followed up for more than 5 years.

General assessment of postoperative symptoms in all patients can be seen in Table 7.

**Table 7:** Postoperative assessment of symptoms.

	Asymptomatic		Better		Unaltered		Worse	
	n	%	n	%	n	%	n	%
Nasal obstruction	37	59,6	18	29,0	6	9,6	1	1,6
Postnasal dripping	32	51,6	22	35,4	6	9,6	2	3,2
Facial pressure	55	88,7	4	6,4	3	4,8		
Nasal secretion	31	50,0	23	37,1	6	9,6	2	3,2
Sore throat	51	82,2	10	16,1	1	1,6		
Facial pain	61	98,3	1	1,6				
Cough	43	69,3	11	17,7	4	6,4	2	3,2
Fatigue	55	88,7	5	8,1	2	3,3		
Altered olfaction	53	85,5	7	11,3	2	3,2		

In Table 8, we can evidence the significant symptomatic improvement of the main complaints of patients with AFRS and fungal ball, comparing its presence pre and postoperatively.

**Table 8:** Progression of symptoms of patients with fungal ball and allergic fungal rhinosinusitis after surgical intervention.

	RSFA		Fungal ball	
	before	after	before	after
nasal obstruction*	100	23	90	4
postnasal dripping*	100	23	84	10
nasal secretion*	95	23	97	10
sore throat*	77	14	58	13
cough*	68	9	13	–
Altered olfaction*	50	5	20	–

* p < 0.001 for chi-square test.

When we compare the symptoms of both groups after the surgical procedure, using the same scale of scores, we evidence that the symptoms in patients with fungal ball are significantly smaller than the ones with AFRS, regardless of the test used. Parametric test (t test for matched samples) showed p < 0.0001 with CI 95% from 2.38 to 3.89; in Wilcoxon t test (non-parametric test for matched samples) we found p < 0.0001, value that was repeated in ANOVA for repetitive measurements.

Sixteen patients (25.8%) presented recurrence and 14 required surgical reintervention: 9 because of nasal polyposis, 4 because of fungal recurrence and 1 for purulent rhinorrhea non-responsive to pharmacological treatment. The interval between the primary surgical procedure and reintervention ranged from 10 months to 11 years.

Eleven patients (45.8%) with AFRS required surgical reintervention (Figure 7): 10 (41.6%) owing to recurrence of the disease and polyposis and one case because of contralateral disease. Only 3 patients (9%) with fungal ball required new surgery. The number of reinterventions was significantly higher (p < 0.002) in patients with AFRS than those with fungal ball.

As to use of postoperative drugs, 7 patients (11.2%) (3 indolent infections and 4 with AFRS) were treated with oral antifungal agents, 56 (90.3%) were treated with antibiotics, and 55 (88.7%) were treated with nasal lavage with saline solution. For patients with AFRS, oral corticoids were prescribed for 83% of the patients and topical nasal corticoids were prescribed to 87.5%.

## DISCUSSION

Approximately 300 species of fungi were documented as having caused diseases in human beings 5 and 90% of the infections are attributed to few dozens of species 6. Most fungi are exogenous, they exist in the soil, in water and organic debris. Most mycoses are caused by little fungi that are part of the normal flora, such as candidiasis, or are highly adapted to survive in the human body, such as dermatophytosis^6^.

FRS, despite being a little different, is a disease that produces significant morbidity, which may lead to death. Symptoms are easily overlapping with other situations, and it is difficult for the physician to recognize and, thus, diagnose it. Assessment and management of these conditions may require the participation of many specialists, including the otorhinolaryngologist, infectologist, microbiologist, and pathologist, providing appropriate diagnosis and treatment, preventing permanent sequelae and reducing mortality.

Incidence and prevalence of fungal infectious are growing with diversification of pathogenic organisms, as a result of the risk factors such as atopy in AFRS, diabetic ketoacidosis in mucomycosis and use of corticoids in candidiasis^7^. Impaired cell immunity in patients with AIDS also contributes to this growth, as all was in patients that use immunosuppressant or cytotoxic drugs to fight against neoplasm or to participate in programs for organ transplantation, which are getting more frequent in daily medical practice^8^.

The diagnosis of AFRS initially requires high degree of suspicion by the physician, because the clinical history and the physical examination per se are rarely conclusive.

Schell^9^ considered important the presence of fungi in the middle meatus and paranasal sinuses so that we can respond to the difficult differentiations between colonization and pathogenicity, and the laboratory methods that are adopted have to be as simple as possible, to that they are accessible in the daily medical routine.

The true incidence of FRS, especially AFRS among patients with CRS, remains unknown^4^. We demonstrated a prevalence of 6.7% of positive cultures for fungi in the series of patients with CRS submitted to surgical procedures, a value greater than that found for Lessa et al.^10^ (p < 0.05, chi-square test) in a study performed in a public hospital in the city of Sao Paulo, which showed incidence of fungi in 31 patients (4.4%) of the 706 cases with CRS operated on between the years of 1995 and 2000. This difference in prevalence may be explained by our interest in identifying fungi, key objective of the study. Katzenstein et al.,^11^ in 1983, in the description of AFRS, found an incidence of 6.2%; Kupfenberg and Bent^12^ estimated that the fungus was the cause of 5% to 7% of the cases of CRS that required surgical treatment; and Yoon et al.,^3^ in South Korea, assessing 510 patients with CRS submitted to maxillary sinus surgery, found 39 (7.6%) cases of FRS histopathologically confirmed.

Quaraishi and Ramadan^13^ estimated the prevalence of fungi in 7%; Shubert^14^, in a bibliographical review of retrospective studies found values between 5 and 10%, coinciding with what was recorded by Houser and Corey^15^ for patients with CRS with surgical indication.

Vennevald et al.^16^, however, had positive culture for fungi in 25.7% of the 117 immunosuppressed patients with non-invasive CRS submitted to paranasal sinuses endoscopic surgery, a fact that can be explained by the immune status of patients. Lebowitz et al.^17^, studied 45 patients with CRS submitted to endoscopic surgical procedures, isolated fungi in 56% of them using traditional laboratory methods, identical to employees that were routinely employed in the processing of samples for pulmonary secretion.

Because they are ubiquitous fungi, contamination of samples by the culture can occur easily during the collection or laboratory processing. Considering the simple identification of fungi does not necessarily confirms the pathogenicity, the diagnosis of a mycotic infection can only be performed when we analyze the set of data of the culture, surgical findings and results of anatomopathological exams.

Marple and Mabry^5^ pointed out the role of fungi in the development and perpetuation of infection in the upper airways and observed that the literature had strong evidence of allergic and non-allergic forms of non-invasive fungal inflammation. We still do not know if these forms of inflammation are independent or interrelated.

Published data in different studies are extremely varied when we analyze the species of fungi identified in patients with FRS. There is a large number of species and an important regional geographical variance of its prevalence. The most frequently reported genders are Aspergillus, Alternatia, Candida, Penicilium, and Fusarium^4^^,^^5^^,^^7^.

We found species of Aspergillus in more than half of the samples described by Manning^2^, which consider this gender as predominant in cases of non-invasive FRS, a fact confirmed by Donald^18^ and Ruppa el al.^19^ Vennewald et al.^16^ found Aspergillus sp. in 23 (67.4%) patients in a series of 34 patients with FRS without affection of immune system and Kupfenberg^20^ in 19 (41.3%) in a series of 49 patients with the same pathology.

The incidence of a determined gender depends a lot on geographical and climatic conditions in which the patient lives, because the presence of a specific fungus in the environment is related with environmental conditions of temperature and air relative humidity^4^.

When identifying fungus as etiological agents of rhinosinusitis, it is important to classify them into type of FRS to be able to plan appropriate treatment and determine the diagnosis, which depends more on the response to host than to a specific type of fungus that causes the infection^9^. Initially, the response may be divides into two basic types: invasive and non-invasive, based on penetration of the fungus in the mucosa. Non-invasive FRS are diagnosed as fungal ball, saprophyte infestation or AFRS 4. Among them, AFRS is the most controversial in the literature. The diagnostic criteria were initially proposed by Katzenstein^11^, with the presence of fungi without invasion of mucosa, mucus-thickness, eosinophilic inflammation and Charcot-Leyden crystals.

In the clinical presentation of patients with the diagnosis of FRS, the predominant symptoms that we found were nasal obstruction (92%), nasal secretion (88.7%), postnasal dripping (82.2%), and cough (69.35%), common to all patients with CRS, thus, it is not important in the suspicion or presence of fungi. The most frequent comorbidity in our series was allergic rhinitis (53.2%), practically the same incidence (62%) marked by Kalinger^21^ in a study with 200 consecutive patients with CRS that were seen by allergists or immunologists. The frequency of bronchial asthma (38.2%) is similar to that found by Manning^2^.

After surgical procedure, patients were classified based on type of FRS according to the criteria adopted by Ferguson^4^, based on surgical, microbiological and histopathological findings.

No occurrence of invasive RFS was identified in our series. Invasive RFS is a situation in which it affects people with severe associated diseases and the diagnosis is normally made in patients that are not hospitalized 4, 8. It always affects immunocompromised patients, patients with AIDS or those that take corticoids or immunosuppressant drugs for the treatment of neoplasms or after organ transplantation. Diabetic patients in ketoacidosis have high risk for invasive disease caused by fungi in Zygomycetes, which prefer acid environment with high concentration of glucose. The absence of invasive RFS in our series can be explained by the origin of patients: they were all outpatients who did not have severe diseases.

Based on these findings, we decided to concentrate on the study of these two groups that are more prevalent, fungal ball and AFRS, comparing the clinical and surgical findings and progression after treatment.

Upon comparing the age of the two groups we found a difference between them, despite the fact that the fungal ball is found in the literature as a situation in which the most advanced age patients are affected^4^^,^^7^^,^^22^.

Preoperative findings of patients with diagnosis of fungal ball, when compared to AFRS, did not show statistically significant difference concerning nasal secretion and obstruction, use of systemic antibiotics, history of allergic rhinitis, and repetitive bronchopneumonia. Patients with AFRS presented significantly greater number of bronchial asthma, intolerance to aspirin and use of topical corticoids, facts that may be explained by the presence of atopy in these patients. Ferguson^22^, in a literature review, had not found differences between the symptoms of patients with fungal ball when compared to patients that have other types of FRS.

The presence of polyps was statistically greater in cases of AFRS, data compatible with that reported by the authors Houser and Corey^15^, who found polyps in all patients with AFRS, and those by Ferguson^22^, who presented 10% of patients with fungal ball. Those with AFRS also had bone erosion and thickness of mucosa that were significantly greater. Houser and Corey^15^ described bone erosion in only 20% of the cases in a literature review.

After they had been individually analyzed, pre and postoperative data were statistically treated and included in a scale of values, enabling global comparison of symptomatology between the two groups. This global comparison of symptoms did not show differences between them, a fact that was reported by Ferguson^4^, Houser and Corey^15^ and Manning^7^.

However, when these symptoms were individually compared, patients with AFRS presented more intolerance to aspirin, bronchial asthma, presence of nasal polyposis and previous use of topical corticoids. These 4 factors, similarly to AFRS, are known to be related to atopy, a characteristic that may explain this difference.

The great difference in polyposis in patients with AFRS is compatible with the findings published in the literature, and their presence is normally reported as diagnostic criteria. Houser and Corey^15^ considered the incidence of polyps in practically 100% of the cases. Kupfenberg and Bent^12^, and Manning^2^ found polyps in all patients of their series, submitted to surgical treatment.

In patients with diagnosis of fungal ball, the presence of polyposis in 2 (6.1%) was similar to that found by Ferguson 22 (10%) in a bibliographical review of 158 cases.

In the symptomatic assessment after the surgical procedure, we observed improvement in all items related with over 90% of the patients, confirming efficiency and benefit of the surgery, similar to the results obtained in patients of CRS^23^^,^^24^.

Using the same scale of values for symptoms, we observed significantly better symptomatic improvement in patients with fungal ball, who had fewer recurrences and required smaller number of reinterventions when compared to AFRS. These results evidenced the recurrent character and the severity of AFRS.

For patients with fungal ball, the level of recurrence (9%) is similar to that found by Ferguson^22^, estimated in the literature review as between 4% and 10%. Manning^7^ and Kupfenberg^20^ considered that the recurrence of fungal ball is rare.

The number of recurrences of AFRS was much greater, and in 45.8% of them they required surgical reintervention, marking the chronic and recurrent characteristic of the disease and its difficult control. The success of treatment with AFRS depends on three steps: to make surgical debridment to remove fungal antigens, allergic mucin and affected polypoid mucosa; to prevent recurrence of fungal growth, and to modify the immune response of host to antigen. Given that we do not always get the appropriate control of allergic symptomatology, we can expect postoperative follow-up with more recurrences.

The literature is quite controversial about the real role of fungi in CRS and we agree with most authors^4^^,^^7^^,^^9^^,^^11^^,^^17^ that consider that the simple identification of the fungus is not enough for diagnosis, which should be performed together with surgical and clinical pathological findings.

There are few questions about the fact that fungi or their protein components may stimulate the respiratory tract through allergic mechanisms mediated by IgE. The literature offers much evidence that explain the allergic and non-allergic forms of non-invasive fungal infections. In the future, we should define how much these forms are interrelated or independent one from the other.

The understanding of immune mechanisms and the way through which we can interfere on them may increase the effectiveness of diagnoses and treatments, reducing the recurrence of the disease, especially in cases of AFRS.

Fungal infection is an emerging problem in daily clinical practice, and its prevalence has increased owing to a large number of chronic diseases resulting from aging of the population and/or from pathological situations that require use of drugs that affect the immune system.

In our study, we tried to define the prevalence of fungal infection in immunocompetent patients, as well as type of clinical presentation of the disease. The knowledge of these facts is important in the clinical suspicion and investigative approach of ENT patients with CRS in our daily practice.

## CONCLUSION

The prevalence of FRS was 6.7% in patients with CRS submitted to paranasal sinuses endoscopic surgery, with predominance of Aspergillus, including the significant amount of Candida, Penicilium and Alternaria.

The type of FRS most frequently detected was fungal ball in more than half of the cases, followed by AFRS, present in more than one third of the patients. The diagnosis of indolent FRS and saprophyte infection was not very frequent. The progression after endoscopic surgery was more favorable in those with fungal ball, which required fewer interventions.

We suggest that new studies are performed to complement the one we had started especially with immunosuppressed patients and those with invasive disease, which were not included in our study.

The increase in our knowledge and understanding of the fungal disease will make us enhance our clinical suspicion, precision of diagnosis and effectiveness of treatment, providing better prognosis to our patients.
